# Upgrading instructions for authors of scholarly journals

**DOI:** 10.3325/cmj.2014.55.271

**Published:** 2014-06

**Authors:** Armen Yuri Gasparyan, Lilit Ayvazyan, Sergey V. Gorin, George D. Kitas

**Affiliations:** 1 Departments of Rheumatology and Research & Development, Dudley Group NHS Foundation Trust (Teaching Trust of University of Birmingham), Russells Hall Hospital, Dudley, United Kingdom; 2 Department of Medical Chemistry, Yerevan State Medical University, Yerevan, Armenia; 3 Head of the Russian Regional Chapter of the European Association of Science Editors; Chief Editor of *International Scientific Researches*, Moscow, Russian Federation; 4 Arthritis Research UK Epidemiology Unit, University of Manchester, Manchester, United Kingdom

Publishing a scholarly article is the final and perhaps most responsible stage of research that takes time and requires enormous effort of all stakeholders of science communication. Reviewers and editors when they evaluate journal submissions and select items for publishing often ground their decisions on the novelty and originality of the topics, validity and power of statistical and other tests, quality of writing, structuring, and formatting each section of the manuscripts, originality and usefulness of graphical materials, and professionalism in analyzing scientific facts and conclusions. The evaluators also consider ethical issues and the adherence of authors to the guidance from editorial associations (1,2). The authors often receive rejection letters pointing to the mismatch between the scope of the journal and the manuscript topic, methodological errors, inappropriate discussion, unjustified conclusions, and poor writing and formatting (3,4). With the increasing flow of journal submissions it is expected that rejection rates will increase further, causing more frustration for inadequately instructed authors.

To better guide authors and avoid wasting the efforts of all those involved in scholarly communications, it is required to regularly revise and upgrade journal instructions, inform authors about the journal’s scope, priority articles, peer review policy, code of publishing ethics, structure and content of different types of accepted articles, in-house style of editing and formatting, and accompanying documents required for each submission (Box 1) (5,6). Properly written, printed, and available online instructions are the keys to successful publishing and indexing in prestigious bibliographic databases. Moreover, records in the instructions are increasingly used as primary data for science communication research, and accurate details of the journal editorial policies and procedures would contribute to the evidence accumulation in the field.

BOX 1 **Main sections of the instructions for authors of scholarly journals**

**Table:** 

Subject areas and specific scope of the journal
Types of published articles and their priority for the journal
Preparation and formatting of all sections of manuscripts, covering letters, and supplementary materials
Research reporting guidelines to consult
Internal and external peer review policy
Online registration and submission guide
Research ethics considerations
Authorship criteria and authors’ contribution details
Conflicts of interest disclosures
Definition of plagiarism and related procedures
Ethical considerations for duplicate (redundant) and secondary publications and retractions
Copyright forms and licenses
Open access models employed
Publication and open access charges

Not all editors of newly launched journals regard the work on the instructions as critically important, and either copy them from other periodicals or prepare abridged versions that often lack details of the scope, priorities, and practised editorial procedures. At the same time, editors of some established journals overlook the importance of updating their instructions in line with the revised recommendations of major editorial organizations, thus hindering the journals’ development prospects.

There are no universally acceptable instructions, encompassing all the necessary points and satisfying specific requirements of each discipline. Journals may choose to endorse their own set of the submission and publishing regulations. Large publishing corporations, such as the BMJ group, Elsevier, and Springer, develop general guidelines for the whole portfolio of their journals, with specific details being added to the online instructions by each periodical.

Journal editors are supposed to develop and revise their instructions in compliance with the recommendations of editorial organizations and in line with the available evidence on appropriate editorial practice. A recent large survey, however, revealed that editors are often reluctant to change or enforce important elements in their instructions, thus creating a publication bias and distorting the scientific evidence accumulation (7). The wide variations in the instructions of evidence-based journals exist despite the decades-long campaign to systematize research reporting and to stick to the guidance of major organizations, such as the International Committee of Medical Journal Editors (ICMJE) and the Enhancing the QUAlity and Transparency Of health Research (EQUATOR) network (1,8,9).

Reluctance to upgrade the instructions primarily disadvantages nonmainstream science and low-impact journals. In a recent survey of the journal instructions of 56 Latin American and Caribbean biomedical journals references to the CONSORT (Consolidated Standards of Reporting Trials) guidance were found in only 7 (13%) journal instructions, while to PRISMA (Preferred Reporting Items for Systematic Reviews and Meta-Analyses) – in just 1 (1.8%) (10). In a study of urology and nephrology journals, listed in the Journal Citation Reports^®^, a positive association was found between impact factor and frequency of referring to CONSORT in the instructions (11).

For high-impact journals, where many reporting guidelines are already mentioned in the instructions, a more pressing issue is that of inaccurate reporting; and it seems to lie with the low enforcement of the available guidelines. An analysis of 258 trials, reported in the *BMJ*, *JAMA*, *Lancet*, and the *New England Journal of Medicine* in 2010, found that the method of randomization was unclear in 37% and that complete adjustments for balancing factors were missing in 74% of trial reports (12). The inaccurate adherence to CONSORT was also apparent in 118 trial reports, recently published in six high-impact rheumatology journals, where an informative account on patient recruitment was missing in 82 (69.5%) reports (13).

## Main sections of the instructions

### Scope and priorities

High-impact general and specialized scholarly journals with a long publishing history and an extensive experience of implementing ethical, research reporting, and writing standards often publicize elaborate instructions. The exemplary instructions of the *New England Journal of Medicine*, *The Lancet, JAMA, Nature Medicine, Annals of the Rheumatic Diseases*, and some other top-tier journals have been in the spotlight of the committed editors and demanding authors for a long time, which allowed to select and publish the most influential and scientifically sound papers in these journals (14).

In the past few decades, the instructions of the most influential journals explicitly prioritized randomized controlled trials, systematic reviews, meta-analyses, and large cohort studies, which contributed to the growth of the journals’ evidence base and impact indicators more substantially than short communications and case reports (15-17). Some high-impact journals completely abandoned case reports to secure more space for “more citable items.” *The Lancet* and other flagship medical journals, on the other hand, continued publishing clinical cases which may have educational points (18). The situation changed in 2013, when the CARE (Case REport) guidelines and related 13-item checklist for accurate reporting of cases were published and widely promoted to encourage the authors to publish more transparent and better structured cases (19,20).

Some editors of top-tier journals limit the number of review articles by soliciting them from eminent authors and advising others to discuss the contents of their reviews with responsible editors prior to submission. Many others, however, encourage the authors to submit narrative reviews, which are still the most-read and citable items (21).

### Format and references

Once the decision to submit a manuscript is made, it is worth checking the formatting and referencing instructions of the target journal. Accurately composed textual and graphical materials, references, and related supplementary materials may shorten the time of in-house editing of accepted manuscripts. The journal instructions have to cover the essential writing and formatting details but avoid extensive technical descriptions in this section, particularly at the expense of other sections, and given the fact that most online periodicals have standardized digital formatting. The updated instructions should primarily draw attention to unethical and irrelevant references which may be used to artificially boost the citation indexes and damage the journal's reputation as an unbiased and ethical source of information. This is particularly important for journals from small, nonmainstream science, and highly specialized professional communities, where auto-citations are common and are not always relevant or ethical (22,23). A related link to the San Francisco Declaration on Research Assessment (DORA) can be useful in this section of the journal instructions (24). The Declaration, which was initiated by the American Society for Cell Biology (ASCB) in 2012, has been endorsed by 10 668 individuals and 467 organizations so far. It strongly advises the researchers to avoid the “citation games,” cite primary sources rather than reviews, and appropriately interpret various citation indexes, which by no means can be used as the proxies of the journal scientific merits.

### Peer review

The majority of indexed journals assess the quality, originality, and integrity of the submissions through the peer review. Its models, types of submissions forwarded to external reviewers, timelines, number of evaluators per manuscript, and the overall quality vary between journals from different disciplines and geographic regions (25-27). The journal editors, particularly those who declare the adherence to the recommendations of the Committee on Publication Ethics (COPE), should transparently describe the type of peer review (single-, double-blinded, open, or post-publication), any difference between reviewing various types of manuscripts, regular, and special issues, confidentiality, and disclosure of conflicts of interest in the process of evaluation (28). Additionally, they should give their authors the opportunity to list the most and least desirable reviewers for fairer evaluations. Transparency and other ethical points, however, are not always taken into consideration at the writing or upgrading the peer review section in the journal instructions. For example, we searched through the online instructions for authors of 44 rheumatology journals that are listed in the SCImago Journal & Country Rank database (as of April 10, 2014), and found that the peer review model and the number of external reviewers were mentioned in only 10 (22.7%) and 11 (25%) journals, respectively (Table 1). The journal impact indicators did not influence the completeness of the peer review descriptions.

**Table 1:** Statements on peer review in the online instructions of rheumatology journals listed in the SCImago database*

Rank	Abbreviated journal titles	H index	2-y JIF	Peer review type	Open review option	N of reviewers	Statistical reviewer involved	Policy for editors’ submissions	Author suggest reviewers
1	*Arthritis Rheum*	211	7.477	?	?	?	-	+	+
2	*Ann Rheum Dis*	132	9.111	?	+	≥1	+	+	-
3	*J Rheumatol*	124	3.258	?	?	1-3	-	-	-
4	*Rheumatology*	106	4.212	SB	-	?	-	+	+
5	*Arthritis Res Ther*	84	4.302	?	?	2	-	-	-
6	*Arthritis Care Res*	82	3.731	?	-	?	-	-	+
7	*Semin Arthritis Rheum*	73	3.806	?	-	?	-	-	+
8	*Clin Exp Rheumatol*	62	2.655	-	-	-	-	-	-
9	*Rheum Dis Clin North Am*	61	2.096	-	-	-	-	-	-
10	*Nat Rev Rheumatol*	52	9.745	?	-	3	-	-	-
11	*Joint Bone Spine*	43	2.748	?	-	?	-	-	-
12	*Rheumatol int*	43	2.214	DB	-	?	-	-	-
13	*BMC Musculoskelet Dis*	41	1.875	-	+	2	-	-	+
14	*Curr Rheumatol Rep*	37	-	?	-	?	-	-	-
15	*Z Rheumatol*	31	0.450	?	-	?	-	-	-
16	*J Clin Rheumatol*	29	1.183	?	-	?	-	-	-
17	*Rev Rhum (Edition Francaise)*	28	-	?	-	?	-	-	-
18	*Bull NYU Hosp Jt Dis*	26	-	?	-	?	-	-	-
19	*J Musculoskelet Pain*	25	0.328	DB	-	?	-	-	+
20	*Reumatismo*	13	-	?	-	?	-	-	-
21	*Biologics*	12	-	SB	-	?	-	-	-
22	*Int J Rheum Dis*	12	1.65	?	-	2	-	-	-
23	*Musculoskelet Care*	12	-	DB	-	?	-	-	-
24	*Acta Reumatol Port*	10	0.695	?	-	?	-	-	-
25	*Pediatr Rheumatol*	10	1.47						
26	*Rev Bras Reumatol*	10	-	?	-	?	-	-	-
27	*Akt Rheumatol*	9	0.097	?	-	?	-	-	-
28	*Curr Rheumatol Rev*	7	-	SB	-	3	-	-	-
29	*Reumatologia*	7	-	?	-	?	-	-	-
30	*Reumatol Clin*	7	-	?	-	?	-	-	-
31	*Ceska Revmatol*	6	-	?	-	?	-	-	-
32	*Int J Clin Rheumatol*	6	-	DB	-	≥3	-	-	+
33	*Indian J Rheumatol*	5	-	?	-	≥2	-	-	-
34	*Autoimmunity Highlights*	3	-	?	-	?	-	-	-
35	*Int J Adv Rheumatol*	3	-	?	-	?	-	-	-
36	*Open Access Rheumatol*	3	-	SB	-	?	-	-	-
37	*Open Rheumatol J*	3	-	?	-	?	-	-	+
38	*Reumatol Clin Supl*	3	-	?	-	?	-	-	-
39	*Rev Rhum Monograph*	3	-	?	-	?	-	-	-
40	*Turk J Rheumatol*	3	0.172	DB	-	2	-	-	-
41	*Semin Fund Esp Reumatol*	2	-	?	-	?	-	-	-
42	*Ther Adv Muskuloskelet Dis*	2	-	SB	+	≥2	-	-	+
43	*Rheumatol Rep*	1	-	?	-	?	-	-	-
44	*Open Arthritis J*	0	-	?	-	?	-	-	+

*Data are obtained from the SCImago Journal & Country Rank database, the Journal Citation Reports 2013 (2-Year Journal Impact Factors [2-Y JIF]), and the instructions to authors available on the journal websites as of April 10, 2014. SB – single-blind; DB – double-blind.

Generally, the journal submissions pass initial checks by the editorial staff, and a small number of well-written and formatted items enter the peer review with one or more external reviewers, whose identities are often masked to the authors to avoid conflicts (single-blind review). For reviews in journals of small professional communities, it is reasonable to mask the identities of both reviewers and authors to avoid any personal bias (double-blind review). In case of open review, which is becoming popular, the identities of both reviewers and authors are unmasked, and comments are open to the public. Some journals invite statistical reviewers to assess the reliability and power of statistical analyses. The external reviewers are usually chosen by the editors based on the reviewers’ academic credentials, publication records in prestigious databases, and previous contributions to the same journal, among other criteria (27). Some editors give their authors the option to name potential reviewers in the covering letters without an obligation to stick to the authors’ preferences. Large, interdisciplinary, and international journals with published impact factors usually have a well-established database of actively contributing reviewers, whereas newly launched and highly specialized ones often struggle to find skilled reviewers and to get constructive comments (29). In an attempt to better inform the authors, the journal editors may survey the perceptions of the peer review, its timeliness, and overall quality, and display the results online (30).

### Authorship

The updated guidance about authorship is another critical component of the instructions, which adds to the accuracy and transparency of research reporting (31). Several surveys of large samples of biomedical journal instructions revealed the absence of explicit statements on scientific authorship in 35%-85% of them (31-33). A comparative study of periodicals from various subject categories demonstrated that adherence to the ethical norms of the editorial associations, and particularly to their authorship policy, is worst in the social sciences and arts and humanities (34).

Following much debate over the ethics of research reporting, the 2nd World Conference on Research Integrity issued one of the major documents in the field – the Singapore Statement on Research Integrity (35), which was endorsed by a number of publishers, some of whom uploaded related links on their websites (36-38). The Statement emphasized honest, responsible, and professional reporting at all stages of the research and writing. Two of its points referred to the authors’ responsibility for all their scientific communications and for acknowledging contributions of all those involved in the manuscript writing. The revised in 2013 recommendations of the ICMJE defined the responsibility for the integrity of all parts of the manuscripts as the fourth obligatory criterion of authorship, which was added to the previous three criteria (ie, substantial contribution to the research work, its revision, and final approval for publication) (39). The renewed recommendations also defined non-author contributions, which warrant acknowledgments, but often get the inappropriate authorship credits or are left completely unappreciated (eg, research supervision, technical and language editing, and proofreading) (40,41). Consequently, related revisions in the biomedical journal policies were encouraged globally to prevent unethical practice and to ensure the integrity of the whole process of science communication (42).

In practice, however, not all journals have revised the authorship criteria in their instructions. Such revisions will probably take more time and will require promoting the awareness of the authorship issues. As an example, we scanned online instructions of 44 rheumatology journals and found that statements on authorship were present in only 13 (29.5%) journals (Table 2). Of these, 6 were top-tier journals in the field, with the *h*-index ranging from 82 to 211. A specific reference to the renewed four criteria was present in only 8 (18.2%) instructions, while 6 (13.6%) contained general links to the main webpage of the ICMJE recommendations without a direct link to the authorship criteria.

**Table 2:** Statements on authorship criteria in the online instructions of rheumatology journals listed in the SCImago database*

Rank	Abbreviated journal titles	H index	2-y JIF	Authorship criteria listed	Updated ICMJE criteria (2013) mentioned
1	*Arthritis Rheum*	211	7.477	+	NA
2	*Ann Rheum Dis*	132	9.111	+	+
3	*J Rheumatol*	124	3.258	+	NA
4	*Rheumatology*	106	4.212	+	+
5	*Arthritis Res Ther*	84	4.302	+	+
6	*Arthritis Care Res*	82	3.731	+	NA
7	*Semin Arthritis Rheum*	73	3.806	NA	NA
8	*Clin Exp Rheumatol*	62	2.655	NA	NA
9	*Rheum Dis Clin North Am*	61	2.096	NA	NA
10	*Nat Rev Rheumatol*	52	9.745	NA	NA
11	*Joint Bone Spine*	43	2.748	NA	NA
12	*Rheumatol int*	43	2.214	NA	NA
13	*BMC Musculoskelet Dis*	41	1.875	+	+
14	*Curr Rheumatol Rep*	37	-	NA	NA
15	*Z Rheumatol*	31	0.450	NA	NA
16	*J Clin Rheumatol*	29	1.183	NA	NA
17	*Rev Rhum (Edition Francaise)*	28	-	NA	NA
18	*Bull NYU Hosp Jt Dis*	26	-	NA	NA
19	*J Musculoskelet Pain*	25	0.328	+	+
20	*Reumatismo*	13	-	NA	†
21	*Biologics*	12	-	+	+
22	*Int J Rheum Dis*	12	1.65	NA	†
23	*Musculoskelet Care*	12	-	NA	NA
24	*Acta Reumatol Port*	10	0.695	NA	NA
25	*Pediatr Rheumatol*	10	1.47	+	+
26	*Rev Bras Reumatol*	10	-	NA	†
27	*Akt Rheumatol*	9	0.097	NA	NA
28	*Curr Rheumatol Rev*	7	-	NA	NA
29	*Reumatologia*	7	-	NA	NA
30	*Reumatol Clin*	7	-	+	NA
31	*Ceska Revmatol*	6	-	NA	NA
32	*Int J Clin Rheumatol*	6	-	NA	†
33	*Indian J Rheumatol*	5	-	+	NA
34	*Autoimmunity Highlights*	3	-	NA	NA
35	*Int J Adv Rheumatol*	3	-	NA	NA
36	*Open Access Rheumatol*	3	-	+	+
37	*Open Rheumatol J*	3	-	NA	NA
38	*Reumatol Clin Supl*	3	-	NA	NA
39	*Rev Rhum Monograph*	3	-	NA	NA
40	*Turk J Rheumatol*	3	0.172	NA	†
41	*Semin Fund Esp Reumatol*	2	-	NA	NA
42	*Ther Adv Muskuloskelet Dis*	2	-	NA	NA
43	*Rheumatol Rep*	1	-	NA	†
44	*Open Arthritis J*	0	-	NA	NA

*Data are obtained from the SCImago Journal & Country Rank database, the Journal Citation Reports 2013 (2-Year Journal Impact Factors [2-Y JIF]), and the instructions to authors available at the journal websites as of April 10, 2014. ICMJE – International Committee of Medical Journal Editors; NA – not available.

†A link to the ICMJE website is provided without specifically referring to the renewed authorship criteria.

### Conflicts of interest

Editors and publishers, who seek transparency and reliability for their scholarly publications, should instruct authors how and where to disclose potential conflicts of interest (COI). To capture the authors’ COI, they can be asked to fill in specific structured forms or to list financial and nonfinancial conflicts in the online submission system (28). The policy of COI disclosure is particularly important for journals processing submissions on pharmaceutical and other interventions (43). Journals in other specialized areas with specific origin of conflicts may adopt different policies and techniques for the disclosure, and publicize COI if these are judged relevant to the journals’ ethical interests.

In 2010, the ICMJE developed a form for disclosure of potential COI, which was widely endorsed by biomedical journals (44). The authors of the most biomedical journals are now asked to fill and sign the form at the manuscript submission. Nonetheless, a recent study of the instructions of 399 biomedical journals revealed wide variations in their policies for comprehensive disclosure of COI (45). While the most journals mandated the disclosure of the authors’ financial (90%) and nonfinancial COI (70%), only 39% mandated the editors’ disclosures (45). The higher-impact and clinical journals were more compliant with the comprehensive disclosure policy, and mentioned that in the instructions, than the lower-impact and basic science journals. Apparently, not only authors, but also editors, editorial board members, and reviewers have to disclose COI, and referring to the journal-related financial and nonfinancial secondary interests in the instructions may help improve the reliability and transparency of science communication.

## Policy related to plagiarism

Given an “epidemic” of plagiarism in scholarly publications in the past two decades and the availability of software to check for text recycling, the journal editors have tightened their policies against plagiarism and other forms of research misconduct (46). A study of 213 retracted publications, which were indexed in MEDLINE from 1966 to 2008, found that 89 items (42%), mostly from low-income and non-Anglophone countries, were plagiarized (47). Another study of 754 submissions to the *Croatian Medical Journal* in 2009 and 2010 revealed that various sections of 85 (11%) manuscripts, mostly from China, Croatia, and Turkey, had more than 10% text similarity with other published sources (48). These studies highlighted the importance of defining plagiarism in quantitative terms and informing the authors about unacceptable overlaps in different parts of their manuscripts (49).

The definitions and tools to tackle plagiarism differ across journals. *The Lancet*, for example, informs the authors that all reviews and non-research materials submitted to the journal will be checked by CrossCheck^®^ software to exclude any text recycling (50). Other journals consider the threshold of 10% textual similarity as unacceptable (51). Most scholarly periodicals, however, still have not publicized their policies against plagiarism and have not employed software to detect textual overlaps. A study of 399 influential biomedical journals demonstrated that plagiarism was explicitly defined on websites of 224 (56%) journals, while its screening was practiced by only 112 (28%) journals (52). The experts also expressed concerns that the currently available software cannot distinguish plagiarized graphical materials, translated texts, and ideas and reveal the intentions of the misconduct, pointing to the need for stepping up the journals’ anti-plagiarism policies and defining all instances of “major and minor” plagiarism (53,54).

### Duplicate and secondary publications

Publishing original, “first-hand” information is a top priority for journals contributing to the growth of evidence base. The duplicate (redundant) publication of research studies and case reports in such journals may distort the records in bibliographic databases and affect the reliability of systematic reviews with secondary qualitative and quantitative analyses (55). Processing narrative reviews, essays, editorials, and guidelines, which have been published elsewhere wholly or partly, in the same or other languages, wastes the reviewers', editors', and publishers' resources. The journal editors may, however, consider some practice guidelines, opinion pieces, news notes, and historical papers for simultaneous or secondary publication. In such exceptional cases, the distribution of scholarly information through more than one publishing outlet is aimed to serve interests of professionals from different regions and language environments. Cross-links to simultaneous or primary publications should be provided in such cases to allow correct indexing of the secondary items. The journal instructions can provide links to the available definitions of acceptable overlapping publications and to the editorial actions, which will follow in case of a violation of the ethical submission and publication norms (56). These links are essential for the most nonmainstream science and newly launched journals (57-59). At the manuscript submission, the authors should be advised to acknowledge in their covering letters any overlap with related submissions and publications (eg, conference abstracts, presentations, full papers, book chapters, images) (60).

### Open access, copyrights protection, and publication charges

In the past decade, many traditional journals switched to the open-access publishing model and related amendments in their instructions were followed (6). The open-access movement introduced major changes in the copyrights policies, archiving in digital libraries, and institutional repositories, and determined the choice of target journals by authors (61-63). It also lifted restrictions for the re-use of published sources, and gave a boost to the proliferation of open-access journals with varying article processing, publishing, and archiving fees. Some open-access journals took advantage of the movement and aimed at financial profits at the expense of ethical norms, which made experts to express concerns over the corruption of science publishing (64). For most low-quality journals, it became a common practice to upload the authors' versions of the articles on the journal webpages for a certain fee (65). Some of these journals with soft quality control even managed to get indexed by prestigious databases and archived their contents in PubMed Central and other permanent portals, which posed a threat to established standards of scientific evidence accumulation. In an attempt to tackle this issue and to advise the authors against submitting their papers to the corrupted publishing outlets, Jeffrey Beall, a librarian from the University of Colorado Denver, USA, set his personal blog, which currently lists 477 “predatory” publishers and 303 standalone journals (66). The Beall's list expands each year, and only a few, initially listed publishers, have been removed from the list after amending their editorial procedures and financial policies. As a prime example, Dove Medical Press (New Zealand) was listed as a “predatory open-access publisher” in 2012 and removed from the list a year later (67). The publisher clearly mentioned in the instructions about employing Creative Commons Attribution Non-Commercial licenses (CC-BY-NC), institutional membership fees, and up to 100% waivers for authors from low-income countries.

The whole concept of open-access publishing is now moving toward a comprehensive access with visibility of the journals in the digital databases such as PubMed Central. Journal instructions should inform the authors whether the publication charges are directed to the digital archiving or whether there are charges for color printing and distribution of reprints. The journals may survive the global competition and improve further by fairer, diversified, applicable to the local circumstances, and transparent financial policies (68,69), which should be clearly communicated to the authors at the manuscript submission.

## Conclusion

Journal instructions are important and need to be properly structured, linked to the available guidelines from editorial associations, and regularly revised and enforced to avoid unethical and erroneous publications. Each scholarly journal, be it a standalone or a part of a major publishing corporation, has to develop its own guidance for a specific group of authors. The web location and revision date of the instructions have to be clearly marked to inform not only the authors, but also reviewers, indexers, and those involved in related research studies. With the current digitization trends, many journals minimize the descriptions of technical formatting and provide more space for ethical guidance and policy statements of the global editorial associations (70). Generally, higher-impact journals seem to have more upgraded instructions than lower-impact ones, though variations may exist across different geographic regions and subject categories. For example, in the case of rheumatology journals, discussed in this article, transparent descriptions of peer review and updated authorship criteria were missing in some top-tier journals. But even properly upgraded and comprehensive instructions, and particularly in high-impact journals, may not be sufficient for the authors’ compliance with the guidance (11-13). Regularly checking and reporting counts and geographic distribution of downloads of the instructions may provide valuable information, warranting properly addressed enforcements of the guidance.
